# Conference Proceedings – 5th International Conference on Molecular Diagnostics and Biomarker Discovery (MDBD 2020): Towards digital healthcare technology: Kota Bharu, Malaysia. 17-18 November 2020

**DOI:** 10.1186/s12919-020-00201-4

**Published:** 2020-12-02

## S-1 Applications of IoT in healthcare and life sciences

### Lee Ken Yong (ken-yong_lee@keysight.com)

#### Keysight Technologies Malaysia Sdn Bhd. Bayan Lepas, Penang, MALAYSIA

From the International Conference on Molecular Diagnostics & Biomarker Discovery 2020 (MDBD2020)

Kota Bharu, Malaysia. 17 – 18 November 2020

**Background**

Healthcare and life sciences industry has seen remarkable advancements in recent years due to the increasing adoption of IoT technologies. IoT has revolutionized the realm of healthcare by providing effective preventive care and diagnosis, remote patient monitoring, and outcome-based treatment to patients.

This seminar gives an overview of digital transformation in the healthcare and life sciences. We will discuss the technology trends and the 5Cs of IoT, namely connectivity, continuity, compliance, coexistence and cybersecurity. You will also discover how the new AI-powered test automation empowers customers to achieve true end-to-end automation for the best possible patient care at every interaction.

## S-2 Rapid diagnostic tools for infectious diseases

### Mohammed Zourob (mzourob@alfaisal.edu)

#### Alfaisal University, Al Zahrawi Street, Al Maather, Al Takhassusi Rd, Riyadh 11533, SAUDI ARABIA

From the International Conference on Molecular Diagnostics & Biomarker Discovery 2020 (MDBD2020)

Kota Bharu, Malaysia. 17 – 18 November 2020

**Background**

According to the World Health Organization (WHO), infect 5-10% of the world population resulting in 3 to 5 million cases of severe illness and 290,000 to 650,000 annual deaths from viruses and respiratory pathogens. Early diagnosis and therapeutic intervention can ameliorate symptoms of infection and reduce mortality. The conventional diagnosis of viral infections has evolved over the years with diverse approaches, however, there are inherent short comings associated with these testing. There is an urgent need for the rapid and low-cost diagnostic assays, due to the enormous annual burden of the influenza diseases and associated mortality.

In this presentation, we will cover our recent results in the development of use of flexible diagnostic tools such as Q-tips, and flexible polymers as low-cost and easy to use colorimetric biosensing platforms. These swabs serve as sample collection, analytes pre-concentration as well as sensing tool. These platforms were tested for various bacteria e.g. *E. Coli, Staphylococcus aureus, Campylobacter, Brucella*, and viruses e.g. flu viruses, COVID-19 and MERS-CoV.

The assay can be performed in field and at the patient bed’s side by minimally skilled personnel without the need for instrumentation. Cross-reactivity assays did not show binding with other common respiratory viruses and pathogens. The detection limit for these viruses and pathogen equivalent to gold standard techniques but can be achieved within minutes.

## S-3 Future of personalized medicine in infectious diseases

### Maha AlMozaini (mozaini@kfshr.edu.sa)

#### King Faisal Specialist Hospital & Research Centre, KSA

From the International Conference on Molecular Diagnostics & Biomarker Discovery 2020 (MDBD2020)

Penang, Malaysia. 17 – 18 November 2020

**Background**

During our daily practice in infectious diseases, we are always reminded that the illness presentation and associated symptoms can vary from one individual to another despite the identical causative pathogens. This variability’s reflect an amalgamation of several factors and most importantly genetic predisposition and polymorphisms. To date, the best-studied application of personalized medicine is in the field of oncology but few or almost nothing regarding infectious disease. Yet there is strong evidence that a precision/personalized medicine approach in infectious diseases would be very effective. In fact, evidence show from your daily practice in infectious diseases; such as in the case with COVID-19 infected individuals, what are the factors that allow one patient to control infection, but another to fall gravely ill?. Hence predictive models are one approach to control infections. In this presentation, we will briefly discuss some of those related projects that are actively ongoing in our institution.

## S-4 Utilization of network analysis for cancer biomarkers discovery

### Wan Fahmi Wan Mohamad Nazarie (wanfahmi@ums.edu.my)

#### Faculty of Science and Natural Resources Universiti Malaysia Sabah 88400 Kota Kinabalu, Sabah, MALAYSIA

From the International Conference on Molecular Diagnostics & Biomarker Discovery 2020 (MDBD2020)

Kota Bharu, Malaysia. 17 – 18 November 2020

**Background**

Cancer is usually planned as a model case of a system biology disease or network disease because of its exceptional heterogeneousness and complexness. There is an essential need for appropriate biomarkers from system-level analyses for cancer detection and/or outcome prediction. Methods focused on the application of omics data into networks have the ability to revolutionise cancer biomarker recognition. It is undeniably important to unravel the biochemical networks underlying cancer in order to explain the disease's molecular pathways and identify effective biomarkers. The networks designed for cancer biomarker discovery focused on various omics level data are identified and illustrated in this talk from recent advances in the area. Precision cancer medicine promises to use genomics knowledge to customise treatment decisions for patients. Biological processes, however, are complex and patients can be differentiated not only by particular genetic changes in their tumours, but also by more ambiguous associations between these changes. In addition to the therapeutic actionability of human mutations, system biology and, more precisely, network analysis provide a foundation for advancing precision medicine. It is important for understanding the dynamics of cancer to find the network biomarkers of cancers and the study of cancer-driving genes involved in these biomarkers. Gene clusters in networks of co-expression are widely known as functional groups. This work is focused on the premise that the dense clusters or communities in cancer patients’ gene co-expression networks which constitute functional units regarding the initiation and progression of cancer.

## S-5 Glycoproteomic in early cancer diagnosis

### Low Ley Hian (leyhian@venn.bio)

#### InterVenn Biosciences Venn Biosciences Sdn. Bhd. G-LC-Room 1, Enterprise 4 Technology Park Malaysia, Bukit Jalil 57000 Kuala Lumpur, MALAYSIA

From the International Conference on Molecular Diagnostics & Biomarker Discovery 2020 (MDBD2020)

Kota Bharu, Malaysia. 17 – 18 November 2020

**Background**

Glycans (carbohydrates) are one of four major classes of biomacromolecules present in all lifeforms. Driven by complex biosynthetic pathways involving genetic and environmental factors, glycans are adaptive to environmental changes. Contrary to other post-translational modifications that typically act as on/off switches, glycosylation dynamically affects protein structure and function. Aberrant glycosylation of proteins has been implicated in key steps of disease biology, including the hallmarks of cancer as well as inflammation cascades associated with autoimmunity and aging. Historically, glycoproteins have been analytically and computationally challenging to study at scale. Unlike DNA, RNA, and proteins, glycans are structurally complex and their synthesis is not template-based. InterVenn has built a platform that overcomes these challenges.

InterVenn harnesses the predictive powers of artificial intelligence combined with cutting edge mass spectrometry to discover clinically relevant biomarkers that can only be revealed by high-resolution analysis of the glycoproteome. We have identified distinct glycopeptide signatures at various stages for diseases including cancer, autoimmune and neurodegeneration. The data InterVenn produces is used for companion diagnostics, prognosis, treatment selection, patient enrichment, recurrence monitoring and biomarker discovery.

InterVenn glycoproteomic based classifiers show specificity and sensitivity in 95%+ range for indications including Ovarian, CRC, Prostate, Pancreatic, Lung, Liver, Melanoma and many other indications.

## S-6 Artificial intelligence & machine learning - moving from theory to clinical practice

### Ying Poh Chan (ypchan@oppstar.com.my)

#### Oppstar Technology Sdn. Bhd., Crest Place.sains@usm 10, Persiaran Bukit Jambul, 11900 Bayan Lepas Penang, MALAYSIA

From the International Conference on Molecular Diagnostics & Biomarker Discovery 2020 (MDBD2020)

Kota Bharu, Malaysia. 17 – 18 November 2020

**Background**

Machine learning is part of everyday life for many places around the world today, from navigation apps to internet shopping, and widely used in other industries, such as retail and banking. But it isn’t routine in healthcare because of the complexity and limited availability of data, and the lack of access to highly skilled data scientists required to turn that data into meaningful improvements. However, in the last 3 years, Machine learning in medicine has made quite a number of big headlines. FDA is also continuously making adjustment to the conventional regulation to start approving AI/ML-driven medical devices and solutions. As machine learning evolves and getting high adoption rate in medical field, healthcare service providers have better information to provide patients. Predictive algorithms, machine learning and advanced analystics can give us a better predictive model of mortality that doctors can use to educate patients at the point of care.

## S-7 Spurring Innovation in Digital Health

### Rajifah Ramli (rajifah@data8.my)

#### DATA8 Sdn Bhd, Kuala Lumpur, MALAYSIA

From the International Conference on Molecular Diagnostics & Biomarker Discovery 2020 (MDBD2020)

Penang, Malaysia. 17 – 18 November 2020

**Background**

This session will look at the trend in digital health globally. What are the focus areas and their development, in relation to technology, acquisition & INVESTMENT? Development in Malaysia will also be highlighted, including opportunities to get started in digital health.

## O-1 Invasive fusariosis in a burn patient: a case report

### Nur Ain Che Azmi^1^, Nik Haszroel Hysham Nik Hashim^1,2^

#### ^1^Department of Medical Microbiology and Parasitology, School of Medical Sciences, Universiti Sains Malaysia, Health Campus, 16150 Kubang Kerian, Kelantan, Malaysia; ^2^Hospital Universiti Sains Malaysia, Universiti Sains Malaysia, Health Campus, 16150 Kubang Kerian, Kelantan, Malaysia

##### **Correspondence:** Nik Haszroel Hysham Nik Hashim (haszroel@usm.my)

From The International Conference on Molecular Diagnostics & Biomarker Discovery 2020 (MDBD2020)

Kota Bharu, Malaysia. 17 – 18 November 2020

**Background**

*Fusarium* species is one of the various non-Aspergillus molds that can cause an invasive infection that can be life-threatening. However, it may be underestimated as there is a lack of a readily available, cheap, and accurate test for detecting non-Aspergillus molds. The clinical laboratory still relies on the conventional method of culturing the clinical sample on culture media before identification. This process is insensitive and slow and delays the management of the patient.

**Case report**

An elderly patient with multiple underlying medical conditions had acquired flame burn injuries while burning out rubbish at home. Physical examination revealed 52% mixed partial and full-thickness burn over bilateral upper limbs, lower limbs, trunk, and face, as well as inhalational injury. Tissues and blood cultures that were sampled both intra- and post-operatively revealed *Fusarium* spp. Despite given various antimicrobial and antifungals in the ICU the patient deteriorated with multiple infections setting in and eventually succumbed to septic shock with multiorgan failure.

**Conclusion**

Burn patients are susceptible to infection by various opportunistic pathogens. This case report highlights a rare pathogen which may often be undiagnosed. The angio-invasive quality of this mold may be life-threatening. Clinicians need to be more alert of the less common opportunistic pathogens that can infect burn patients.

**Consent to publish**

The patient is deceased. Unfortunately, we were unable to contact the next of kin to get consent for publication. There are no identifiable details of the individual reported within the manuscript.

## O-3 β-globin gene construct with CD26/IVS1-1 mutation mimicking HbE/β-thalassemia

### Nur Atikah Zakaria^1^, Abdul Aziz Mohamed Yusoff^2^, Rosnah Bahar^1^, Shaharum Shamsuddin^3,4^, Ridhwan Abdul Wahab^5^ and Muhammad Farid Johan^1^

#### ^1^Department of Haematology, School of Medical Sciences, Universiti Sains Malaysia, Kelantan, Malaysia; ^2^Department of Neurosciences, School of Medical Sciences, Universiti Sains Malaysia, Kelantan, Malaysia; ^3^School of Health Sciences, Universiti Sains Malaysia, Kelantan, Malaysia; ^4^Universiti Sains Malaysia (USM)-RIKEN Interdisciplinary Collaboration for Advanced Sciences (URICAS), Penang, Malaysia; ^5^Department of Biomedical Sciences, Kulliyyah of Allied Health Sciences, International Islamic University Malaysia, Pahang, Malaysia

##### **Correspondence:** Muhammad Farid Johan (faridjohan@usm.my)

From The International Conference on Molecular Diagnostics & Biomarker Discovery 2020 (MDBD2020)

Kota Bharu, Malaysia. 17 – 18 November 2020

**Background**

HbE/β-thalassemia is among commonest hemoglobinopathy in many Asian countries and responsible for at least one half of all severe β-thalassemia worldwide. A similar β-globin gene mutation in individuals could lead to differences in phenotypic appearance due to epigenetic influence of the disease. HbE/β-thalassemia patients with CD26/IVS1-1 mutation have been shown to have different methylation status [1]. This study aims to clone the β-globin gene with mutation as in HbE/β-thalassemia patients i.e. CD26/IVS1-1 and potentially become a tool for epigenetic expression study.

**Methodology**

Human β-globin gene (NCBI accession number: NG_059281.1) was synthesized (IDT, Singapore) and cloned into pJET1.2/blunt vector (Thermo Fisher Scientific, USA) and propagated in *E. coli* Top10. DNA sequencing was performed to confirm the orientation of the insert gene within the vector. Site-directed mutagenesis was performed by a conventional polymerase chain reaction. Primer sets used were: (1) pJET1.2; 5’- CTG CTT TAA CAC TTG TGC CTG AAC ACC ATA TCC ATC C-3’ (forward) and 5’- CCT ACA ACG GTT CCT GAT GAG GTG GTT AGC ATA GTT C-3’ (reverse), and (2) CD26/IVS1-1; 5’-GAA GTT GGT GGT AAG GCC CTG GGC AGT TTG GTA TCA AG-3’ (forward) and 5’-CTT GAT ACC AAA CTG CCC AGG GCC TTA CCA CCA ACT TC-3’ (reverse). The amplified DNA fragment with desired mutations was extracted from agarose gel and digested with XhoI and XbaI restriction enzymes, as well as pJET1.2 vector. Digested products were extracted by Nucleospin® Gel and PCR Clean-up Kit (Takara Bio, USA) and ligated together by CloneJET PCR Cloning Kit (Thermo Fisher Scientific, USA). The positive clones were sent for DNA sequencing to confirm the mutations.

**Results and Discussion**

Three exons and two intervening sequences of human β-globin gene were synthesized from nucleotide number 51 to 1608, to mimic the HbE/β-thalassemia in the occurrence of cryptic splice site due to CD26 mutation that cause the abnormal splicing of the transcribed mRNA [2]. Correctly oriented β-globin gene within pJET1.2 vector was amplified into: 212 bp (pJET1.2 forward and CD26/IVS1-1 reverse primers), 1500 bp (CD26/IVS1-1 forward and pJET1.2 reverse primers), and 1674 bp (pJET1.2 forward and reverse primers). HbE/β-thalassemia CD26/IVS1-6 was previously established in HeLa cells and found useful for molecular and proteomic studies. Therefore, the cloning of the β-globin gene with CD26/IVS1-1 in this study is predicted to have the ability to explore the new treatment especially targeting the methylation status of the β-globin gene carrying the HbE/β-thalassemia trait.

**Conclusion**

Cloning of β-globin gene with CD26/IVS1-1 mimicking mutations in HbE/β-thalassemia allows the exploration of various approach in finding the cure for the disease.

**References**

[1] Haiyuni, et al. *LARP2* DNA methylation in transfusion-dependent Haemoglobin E-Beta (HbE/β) and β-Thalassaemia Major patients. Mal J Med Health Sci. 2019; 15:46–53.

[2] Weatherall. Beginnings: the molecular pathology of hemoglobin. In: Provan et al. (eds). Molecular Hematology, UK, Blackwell Publishing, 2010, p. 1–19.

## O-4 Combination of traditional and current techniques of DNA assembly for rapid production and cloning of DNA construct

### Muhammad Azharuddin Azali^1,2^, Salmah Mohamed^2^, Azian Harun^3^, Shaharum Shamsuddin^4,5^ and Muhammad Farid Johan^1^

#### ^1^Department of Haematology, School of Medical Sciences, Universiti Sains Malaysia, Kubang Kerian, Malaysia; ^2^School of Agriculture Science and Biotechnology, Faculty of Bioresources and Food Industry, Universiti Sultan Zainal Abidin, Besut, Malaysia; ^3^Department of Medical Microbiology and Parasitology, School of Medical Sciences, Universiti Sains Malaysia, Kubang Kerian, Malaysia; ^4^School of Health Sciences, Universiti Sains Malaysia, Kubang Kerian, Malaysia; ^5^Institute for Research in Molecular Medicine (INFORMM), Universiti Sains Malaysia, Kubang Kerian, Malaysia

##### **Correspondence:** Muhammad Farid Johan (faridjohan@usm.my)

From The International Conference on Molecular Diagnostics & Biomarker Discovery 2020 (MDBD2020)

Kota Bharu, Malaysia. 17 – 18 November 2020

**Background**

The production of synthetic DNA increasingly necessary especially when dealing with dangerous organism such as scorpion, snakes as well as SARS-CoV-2 virus which causing the current COVID-19 pandemic. *Androctonus australis* Hector is a highly toxic scorpion producing insect toxin AaIT [1]. This gene has been used for creating new recombinant insecticides [2]. Producing this gene synthetically is a necessity so that handling the venomous scorpion is completely avoided. Additionally, the sequence of the DNA can be edited for optimum gene expression.

**Methodology**

The *Androctonus australis* Hector insect toxin (AaIT) construct was synthesised using 10 overlapping oligonucleotides. These oligonucleotides were designed to overlapped with vector as well as other nucleotides and their total lengths are not more than 50 nucleotides. The oligonucleotides were mixed and used at the final concentration of 0.5 μM in assembly PCR. The assembly PCR was carried out at 55 cycles and observed in agarose gel electrophoresis. The PCR product was then enriched in the second PCR. The correct DNA fragment was purified from the gel and then assembled into pCR2.1-TOPO using NEBuilder® HiFi DNA Assembly Master Mix according to the manufacturer’s protocol. Briefly, 9 μl of the purified DNA was mixed with 1 μl vector and 10 μl of master mix and incubated for 4 hours at 50°C before transformed into Top10 *E. coli*. The clones were then screened for the presence of the recombinant plasmid by PCR.

**Results and Discussion**

Assembly PCR produced faint DNA band near the expected region viewed on agarose gel electrophoresis. The second PCR yield a bright band of expected size (307 bp). Colony PCR performed on the transformants yield expected DNA bands confirming that the assembly was successful, and the clones carry the DNA construct in their plasmid.

**Conclusion**

Combination of assembly PCR and new assembly method can be used for rapid synthesis and cloning of the DNA construct from the overlapping oligonucleotides.

**References**

[1] JI SJ, et al. Recombinant scorpion insectotoxin aait kills specifically insect cells but not human cells. Cell Res 2002,12:143–150.

[2] Deng SQ, et al. Application of the scorpion neurotoxin aait against insect pests. Int J Mol Sci. 2019, 20:3467.

## O-5 Effect of palm oil-derived tocotrienol-rich fraction on oxidative stress and inflammatory biomarkers in streptozotocin-induced diabetic rodent model

### Muhammad Zulfiqah Sadikan^1^, Nurul Alimah Abdul Nasir^1^, Igor Nikolayevich Iezhitsa^2^ and Renu Agarwal^2^

#### ^1^Centre for Neuroscience Research (NeuRon), Faculty of Medicine, University Teknologi MARA, Sungai Buloh, Selangor, 47000, Malaysia; ^2^School of Medicine, International Medical University, Bukit Jalil, 57000 Kuala Lumpur, Malaysia

##### **Correspondence:** Nurul Alimah Abdul Nasir (nurulalimah@uitm.edu.my)

From The International Conference on Molecular Diagnostics & Biomarker Discovery 2020 (MDBD2020)

Kota Bharu, Malaysia. 17 – 18 November 2020

**Background**

Diabetic retinopathy (DR) is the second leading microvascular complication of diabetes mellitus (DM). Despite high prevalence, current treatment options for DR are suboptimal and are not curative. Chronic inflammation and oxidative stress (OS) induced by hyperglycaemia are among the key factors responsible for the early development of DR. Alternative therapy that possesses anti-oxidant and anti-inflammatory properties such as tocotrienol-rich fraction (TRF) may provide protection against onset and progression of DR. Hence, in this study, we evaluated the effect of TRF on retinal OS and inflammatory markers in a rat model of streptozotocin-induced DR.

**Methodology**

Nine-weeks old of male Sprague-Dawley rats weighing about 200-250 grams were divided into normal-control (NC) and diabetic rats. Diabetic rats were injected with intraperitoneal injection (IP) streptozotocin (STZ) at 55 mg/kg body weight, whereas NC similarly received IP citrate buffer. Diabetic rats were then further divided into DV (diabetic-vehicle treated) and DT (diabetic-TRF treated) groups. TRF was given orally at a dose of 100mg/kg body weight once daily for 12 weeks in DT, whereas DV and NC similarly received vehicle. Throughout experimental period, body weight and blood glucose level were monitored once a week. After 12 weeks, rats were sacrificed, and retinae were collected for measurement of OS and inflammatory markers. OS markers included malondialdehyde (MDA), reduced glutathione (GSH), superoxide dismutase (SOD), and catalase, whereas inflammatory markers included interleukin-1 alpha (IL-1α), interleukin-6 (IL-6), tumor necrotic factor alpha (TNF-α), interferon gamma (IFN-γ), inducible nitric oxide synthase (iNOS), and monocyte chemoattractant protein-1 (MCP-1).

**Results and Discussion**

Body weight gain in DV and DT was lesser than NC (p<0.001) from week 7 until week 12, but greater weight gain was observed in DT compared to DV (p<0.05) from week 9 until the end of treatment. The blood glucose level in DT was also lower in DT compared to DV (p<0.05) from week 4 until the end of treatment. Higher retinal MDA level was seen in DV compared to NC (p<0.001) and lower retinal MDA level was seen in DT compared to DV (p<0.001). In comparison, lower retinal GSH, CAT and SOD levels were seen in DV compared to NC (p<0.001). However, higher GSH, CAT and SOD levels were seen in DT compared to DV (p<0.05). These observations indicate that treatment with TRF increases antioxidant enzyme activity and reduces retinal OS in diabetic rats. Similarly, expression of retinal inflammatory markers including IL-1β, IL-6, TNF-α, IFN-γ, iNOS and MCP-1 was greater in DV compared to NC (p<0.05). Lower expression of all inflammatory markers was observed in DT compared to DV (p<0.05), and this was comparable to NC.

**Conclusion**

Oral administration of TRF at a dose of 100 mg /kg body weight provides protective effect against DR by reducing retinal oxidative stress and inflammation in streptozotocin-induced diabetic rat.

## O-6 Diagnostic potential of glutamate concentration in distinguishing aggressive potential of breast cancer cells

### Irfan Irsyad Bin Azahar^1^, Ahmad Tarmizi Che Has^2^, Siti Norasikin Mohd Nafi^3^ and Noor Fatmawati Mokhtar^1^

#### ^1^Institute for Research in Molecular Medicine (INFORMM), Higher Institution Centre of Excellence (HICoE), Universiti Sains Malaysia, Health Campus, 16150 Kubang Kerian, Kelantan, Malaysia; ^2^Department of Neuroscience, School of Medical Sciences, Universiti Sains Malaysia, Health Campus, 16150 Kubang Kerian, Kelantan, Malaysia; ^3^Department of Pathology, School of Medical Sciences, Universiti Sains Malaysia, Health Campus, 16150 Kubang Kerian, Kelantan, Malaysia

##### **Correspondence:** Irfan Irsyad Bin Azahar (irfanirsyad@ymail.com)

From The International Conference on Molecular Diagnostics & Biomarker Discovery 2020 (MDBD2020)

Kota Bharu, Malaysia. 17 – 18 November 2020

**Background**

Glutamate, a non-essential amino acid commonly known in its role as neurotransmitter has been revealed to be implicitly involved in carcinoma [1]. Research has shown an abnormal upregulation of glutamate in several carcinomas, particularly breast cancer, with additional reports showing further elevation related to a more aggressive tumor progression [2]. Therefore, the diagnostic potential of glutamate is worth exploring, especially for breast cancer phenotypes that lacks common diagnostic markers. Therefore, this study focused on comparing concentration of glutamate in breast cancer cell lines with different aggressive potential, the weakly aggressive MCF-7, highly aggressive MDA-MB-231 and human epithelial breast cell line, MCF-10A as non-cancerous control.

**Methodology**

The basal endogenous and exogenous glutamate concentration in MDA-MB-231, MCF-7 and MCF-10A was measured using fluorometric glutamate assay. This followed by imaging of glutamate specific fluorescence dye in the cell lines via immunocytochemistry.

**Results and Discussion**

Basal endogenous of MDA-MB-231 is shown to be significantly higher (112.95 μM) compared to MCF-7 (29.540 μM) and MCF-10A (73.590 μM). Exogenous glutamate of MDA-MB-231 after 72 hours was also significantly higher (256.368 μM) compared to MCF-7 (108.961 μM) and MCF-10A (148.641 μM). Both results were supported by immunocytochemistry which shown prominence glutamate specific dyes in MDA-MB-231 compared to the other two cell lines. The study has shown that highly aggressive MDA-MB-231 cells have higher endogenous and exogenous glutamate compared to weakly aggressive MCF-7 and non-malignant MCF-10A.

**Conclusion**

Our data supports the notion of the diagnostic potential of glutamate in distinguishing breast cancer cells vs non-cancerous cells and aggressive breast cancer cells vs weakly aggressive breast cancer cells.

**References**

[1] Stepulak, A., et al. (2014), ‘Glutamate and its receptors in cancer’, *J Neural Transm (Vienna),* 121 (8), 933-44.

[2] Budczies, J., et al. (2015), ‘Glutamate enrichment as new diagnostic opportunity in breast cancer’, *Int J Cancer,* 136 (7), 1619-28.

## O-7 BRAF V600E mutation in ameloblastoma: a systematic review and meta-analysis

### Mohd Nazzary Mamat@Yusof^1^, Ewe Seng Ch’ng^1^ and Nawal Radhiah Abdul Rahman^2^

#### ^1^Oncological and Radiological Sciences Cluster, Advanced Medical and Dental Institute (AMDI), Universiti Sains Malaysia, Kepala Batas, Malaysia; ^2^Craniofacial and Biomaterial Sciences Cluster, Advanced Medical and Dental Institute (AMDI), Universiti Sains Malaysia, Kepala Batas, Malaysia

##### **Correspondence:** Radhiah Abdul Rahman (nawalradhiah@usm.my)

From The International Conference on Molecular Diagnostics & Biomarker Discovery 2020 (MDBD2020)

Kota Bharu, Malaysia. 17 – 18 November 2020

**Background**

Ameloblastoma is a common benign epithelial odontogenic tumour. It is a painless, slow-growing intraosseous tumour, but locally aggressive with a high rate of recurrence [1]. The current treatment relies on surgical treatment. The recent finding of a higher mutation of BRAF V600E in ameloblastoma may offer a better investigation on pathogenesis [2]. This study aims to determine the pooled prevalence of BRAF V600E mutation in ameloblastoma and to correlate BRAF V600E mutation with sociodemographic profile and clinicopathological features.

**Methodology**

Preferred Reporting Items for Systematic Reviews and Meta-Analysis (PRISMA) guidelines were followed [3]. A literature search was conducted in PubMed, Web of Science, Scopus, and Google Scholar databases by using the keywords of “Ameloblastoma”, “BRAF protein”, “Proto-Oncogene Protein B-raf”, “BRAF” and “BRAF V600E”. Meta-analysis proportion was pooled using MedCalc (version 19.4) software. A meta-analysis of associations was performed using RevMan (version 5.4) software. The effect of BRAF V600E on outcome parameters was estimated by odds ratios (ORs) with 95% confidence intervals (CIs) for each study.

**Results and Discussion**

Fourteen articles between the year 2014 and 2020 met the inclusion criteria with a total of 601 ameloblastoma cases. The pooled prevalence of BRAF V600E was 69.4% [95% CI = 59.4–78.6%; P < 0.0001]. The BRAF V600E positive mutation in ameloblastoma was significantly associated with a younger age group (less than 53 years old) [OR = 3.57; 95% CI = 1.97–6.47; P < 0.0001] and in the mandible mutation [OR = 9.80; 95% CI = 5.26–18.27; P < 0.00001]. BRAF V600E positive mutation of ameloblastoma is not significantly associated with gender [OR = 0.91; 95% CI = 0.62–1.33: P = 0.62], histological variants mutation [OR = 0.73; 95% CI = 0.41–1.29; P = 0.28], and recurrences [OR = 0.82; 95% CI = 0.38–1.79; P = 0.62]. In resources limited setting, BRAF V600E mutation testing should be prioritise in young patients. It offers better risk assessment of mandible for surgical managements, also the possibility of targeted adjunctive therapy to preserve the functionality of the jaw.

**Conclusion**

In conclusion, BRAF V600E mutation is notable in the pathogenesis of ameloblastoma. Thus, further studies of targeted therapeutic approach should be encouraged to increase the treatment options, hence having a better treatment outcome.

**References**

[1] McClary, A. C. et al. Ameloblastoma: a clinical review and trends in management. Eur. Arch. Oto-Rhino-Laryngology. 2016, 273: 1649–1661.

[2] Brown, N. A. et al. Activating FGFR2--RAS--BRAF mutations in ameloblastoma. Clin. Cancer Res. 2014, 20: 5517–5526.

[3] Moher, D. et al. Preferred reporting items for systematic reviews and meta-analyses: the PRISMA statement. PRISMA Group. PLoS Med. 2009, 6(7): e1000097.

## O-8 Anti-proliferative activity of andrographolide compound on human glioblastoma DBTRG-05MG cell line

### Nurul Syamimi Othman and Daruliza Kernain

#### Institute for Research in Molecular Medicine (INFORMM), Universiti Sains Malaysia, 11800 Penang, Malaysia

##### **Correspondence:** Daruliza Kernain (daruliza@usm.my)

From The International Conference on Molecular Diagnostics & Biomarker Discovery 2020 (MDBD2020)

Kota Bharu, Malaysia. 17 – 18 November 2020

**Background**

Glioblastoma multiforme (GBM) is one of the rare brain cancer diseases and the major cause of human death. The patient diagnosed with GBM only can survive less than one year due to the limitation of surgery, radiation and chemotherapy that have undesired side effects on patients [1]. There is a need urgent to develop new chemotherapy drugs that can target different mechanisms to treat this disease. Andrographolide (ANDR) is one of the bioactive compound isolated and identified from *Andrographis paniculata* sp. [2]. Its known can inhibit the growth of various cancers by involving a few mechanisms such as oxidative stress, cell cycle arrest, apoptosis, necrosis, proliferation, migration, invasion and other miscellaneous actions [3]. This compound will be used in this study to investigate its cytotoxicity effect on the DBTRG-05MG human glioblastoma cell line.

**Methodology**

The DBTRG-05MG and SVG-p12 were treated with different concentrations of ANDR compound ranging from 0.781-200 μM for 24, 48, and 72 hours. The cytotoxicity effect of ANDR on cell lines was investigated using WST-1 assay. The cell viability and LC_50_ for the cell lines were determined by constructing a graph percentage of cell viability against ANDR concentration using GraphPad Prism software.

**Results and Discussion**

WST-1 assay, the cytotoxicity results showed that LC_50_ values for DBTRG-05MG cell lines were 42.82 μM, 27.34 μM and 7.65 μM at 24, 48 and 72 hours respectively. Based on results obtained, ANDR decreased the percentage of DBTRG-05MG cell viability as compared to control (untreated DBTRG-05MG). ANDR inhibits the cell proliferation of DBTRG-05MG in a dose and time-dependent manner. This result indicated that the ANDR compound has a high potential for drug therapy because it can inhibit the DBTRG-05MG cell line with a lower concentration and not showed any cytotoxicity effect on SVG-p12, normal human glial cells.

**Conclusion**

The results of this study proven that ANDR compound has the potential to be one of the chemoprevention agents for GBM disease because of its ability to inhibit the proliferation of DBTRG-05MG cell lines. More studies are needed to elucidate the molecular mechanism of cell death whether it involves in cell cycle or apoptosis pathway.

**References**

[1] Hanif, F., Muzaffar, K., Perveen, K., Malhi, S. M., & Simjee, S. U. (2017). Glioblastoma multiforme: a review of its epidemiology and pathogenesis through clinical presentation and treatment. Asian Pacific journal of cancer prevention: APJCP, 18(1), 3.

[2] Kandanur, S. G. S., Tamang, N., Golakoti, N. R., & Nanduri, S. (2019). Andrographolide: A natural product template for the generation of structurally and biologically diverse diterpenes. European journal of medicinal chemistry, 176, 513-533.

[3] Islam, M. T., Ali, E. S., Uddin, S. J., Islam, M. A., Shaw, S., Khan, I. N & Găman, M. A. (2018). Andrographolide, a diterpene lactone from Andrographis paniculata and its therapeutic promises in cancer. Cancer Letters, 420, 129-145.

## O-9 The effect of andrographolide on PC3 cell line

### Janany Manimaran and Daruliza Kernain Mohd Azman

#### Institute for Research in Molecular Medicine (INFORMM), Universiti Sains Malaysia, Penang, Malaysia

##### **Correspondence:** Daruliza Kernain Mohd Azman (daruliza@usm.my)

From The International Conference on Molecular Diagnostics & Biomarker Discovery 2020 (MDBD2020)

Kota Bharu, Malaysia. 17 – 18 November 2020

**Background**

Targeted drug therapy is one of the most actively explored field by the researchers in cancer research. There are various bioactive molecules, which is mainly extracted from plants has been showing important cytotoxic effects towards the growth of the cancer cells, for instance Vinca alkaloids from *Cathharathus roseus* G.Don, Podophyllotoxin derivatives from *Podophyllaceae*, Taxanes from *Taxus brevifolia* Nutt and Camptothecin derivatives from *Camptotheca acuminate*. In this study, we will also be focusing on a type of bioactive compound, called Andrographolide, a major diterpenoid extracted from *Andrographis paniculata* which containes three hydroxyl group that plays crucial role in the chemical properties of the primary compound. Andrographolide possess many beneficial therapeutic effects in medicinal field such as anti-inflammatory, anti-thrombotic, anti-bacterial, anti-inflammation and also plays a role as an anti-cancer agent.

**Methodology**

To study the effect of andrographolide on the growth of the cancer cells, PC3 cells, a type of prostate cancer was used as the testing candidate. To evaluate the treatment concentration which is effective to inhibit the growth of the PC3 cell line, a cell proliferation assay has to be conducted. The assay conducted in this study is WST-1 assay which has several benefits compared to the other cell proliferation assays. The WST-assay was carried out in the order of few steps, which was leaded by the seeding of the cancer cells, treatment of the andrographolide compound and finally WST- assay protocol. The cells were seeded in 96 well plate in which 10000 cells per well for 24 hours and treated with various concentration of andrographolide starting from 200 uM of working concentration up to 10 fold dilution for 24 hours, 48 hours and 72 hours. Later, the viability of the cells were measured with the WST-1 via obtaining the OD reading of the viable cells.

**Results and Discussion**

The observation has demonstrated that the 200 uM andrographolide concentration used to treat the PC3 cell lines has showed the highest inhibition rate with the lowest percentage of cell viability as expected. The results were shown to have increasing percentage of cell viability with decreasing the concentration of andrographolide treatment. As this cell proliferation assay was carried out for 24, 48 and 72 hours, the outcome showed that, the lowest IC50 value was obtained from 72 hours which is 47.103 followed by 48 hours (56.812) and 24 hours (159.388). The significance of this data has been proven as it has p value <0.01.

**Conclusion**

The therapeutic effect of andrographolide has been observed with significant OD reading which results in the death of PC3 cell lines. Thus, in a nutshell, the andrographolide does inhibit the growth PC3 cell line, the highest inhibition rate was recorded at 200 uM for 72 hours of the andrographolide treatment.

## O-10 Synthesis, characterization and evaluation of anti- cancer drug delivery potential of engineered iron oxide andrographolide nanoparticles

### Nanthini Ravi and Daruliza Kernain

#### Institute for Research in Molecular Medicine (INFORMM), Universiti Sains Malaysia, Penang, Malaysia

##### **Correspondence:** Daruliza Kernain (daruliza@usm.my)

From The International Conference on Molecular Diagnostics & Biomarker Discovery 2020 (MDBD2020)

Kota Bharu, Malaysia. 17 – 18 November 2020

**Background**

Andrographolide is a bioactive labdane diterpenoid which derived from a traditional medicinal plant known as the *Andrographis paniculata.* The compound has been receiving much attention lately due to its unique properties. Andrographolide has been proven to induce anti proliferative action in human glioblastoma cells and its high lipid solubility permits penetration against Blood Brain Barrier (BBB). Brain cancers are considered as one of the diseases which has a poor prognosis with highest mortality rate. Till recent date, surgery and chemotherapy remains as the most effective treatment tool and over the years, Multidrug Resistance (MDR) has contributed more to the mortality rates. To find solution to the issue, nanocarriers were used due to their size, drug permeability and interaction to the specific receptor of BBB by conjugating andrographolide compound. This study aims to determine the Anti-cancer drug delivery potential of andrographolide Engineered Magnetic Iron Oxide Nanoparticles (AG-IONPs).

**Methodology**

Magnetic Iron Oxide Nanoparticles (MNPs) were synthesized by using reverse co-precipitation method and a brownish red precipitate could be observed. For the preparation of AG-IONP, a homogenized sonicator was used for conjugation. Both the samples were freeze dried and kept in room temperature until further use. For Characterization, UV-Vis was used to determine the plasma resonance shift, Spectrophotometer was used to determine the unbound drug content, Dynamic Light Scattering was used to determine the size and charge of the particles.

**Results and Discussion**

Colour changes to brownish red indicated the formation of IONP. Unbound Drug in the supernatant were measured by using Spectrophotometer at 268nm. The result showed that 65% of andrographolide were present indicating only 35% of the drug was attached to the nanoparticle. The UV-Vis Reading meanwhile showed a peak at 280nm for IONP and a peak at 295nm for AG-IONP indicating a red shift phenomenon. Dynamic Light Scattering result showed that IONP had a major change in the size and the particles formed were smaller. Zeta Potential reading showed that the IONP has a positive charge (18.20mV) meanwhile andrographolide showed a negative charge (-1.38mV).

**Conclusion**

Nanotechnology studies are intensively carried out to determine their ability to act as a drug delivery medium and its effectiveness to overcome Blood Brain Barrier (BBB) by delivering specific amount of drug to the targeted site. Further research is needed to understand the cytotoxicity and molecular mechanism of the AG-IONP to improve their drug delivery ability.

**References**

[1] Casamonti, M., et al. Andrographolide Loaded in Micro- and Nano-Formulations: Improved Bioavailability, Target-Tissue Distribution, and Efficacy of the “King of Bitters.” Engineering. 2019, 5(1), 69–75.

[2] Eugine L, et al. Development, characterisation and toxicity evaluation of nanoparticles of Andrographolide. Int J Pharm Pharm Sci. 2012, 4(SUPPL.1), 497–501

[3] Graverini, G., et al. Solid lipid nanoparticles for delivery of andrographolide across the blood-brain barrier: in vitro and in vivo evaluation. Colloids Surf B Biointerfaces. 2018, 161, 302–313.

## O-11 Aptabody improves binding affinity of DI05 aptamer towards recombinant human ICAM-1 protein

### Khairul Mohd Fadzli Mustaffa^1^, Nik Abdul Aziz Nik Kamarudin^1^, Basyirah Ghazali^1^, Nur Fatihah Mohd Zaidi^1^ and Nurfadhlina Musa^1,2^

#### ^1^Institute for Research in Molecular Medicine (INFORMM), Universiti Sains Malaysia, Penang, Malaysia; ^2^Human Genome Center, School of Medical Science, Health Campus, Universiti Sains Malaysia, 15160 Kubang Kerian, Kelantan, Malaysia

##### **Correspondence:** Khairul Mohd Fadzli Mustaffa (khairulmf@usm.my)

From The International Conference on Molecular Diagnostics & Biomarker Discovery 2020 (MDBD2020)

Kota Bharu, Malaysia. 17 – 18 November 2020

**Background**

Aptamer is a single-stranded nucleic acid in the form of RNA or DNA that has been known to bind to a variety of target molecules through hydrogen bonding and hydrophobic interactions. Previously, our group had selected DNA aptamer against recombinant human ICAM-1 protein. Unfortunately, the binding affinity is low (496 nM). Using antibody-aptamer conjugation concept, this study is aims to describe the proof-of-concept of protein-DNA aptamer conjugate called aptabody to improve the affinity of selected aptamers to the recombinant human ICAM-1 protein.

**Methodology**

In this study, DNA aptamer (DI05) isolated from a previous experiment against recombinant human ICAM-1 (rhICAM-1) was used. In order to improve the binding affinity of the aptamer to target molecules, the isolated aptamer was conjugated to another protein molecule known to not interact with the rhICAM-1 (e.g. green fluorescent protein; GFP) and further characterised by its dissociation constant (Kd) with ELONA.

**Results and Discussion**

We found that, the selected aptamer DI05 when conjugated to the GFP under denatured and undenatured conditions increased 30-fold it affinity towards rhICAM-1 compared to unconjugated DI05 from 496 nM to 108 nM. This study proved that the conjugated aptamer can increase the binding affinity of the aptamer to the target molecule as previous study using an anti-thrombin antibody conjugated with thrombin DNA aptamer also showed increasing its affinity (Kd = 567 pM) compared to antibody alone (Kd = 50 nM) or aptamer alone (Kd = 3.5 nM) [2].

**Conclusion**

This study successfully proved that aptabody aptamer was significantly increase the binding affinity compared to unconjugated/bared aptamer.

**Reference**

[1] Kang S, et al. Improved ligand binding by antibody-aptamer pincers. Bioconjug Chem. 2014, 25(8):1421-7.

## O-12 Antigenicity analysis of Acinetobacter baumannii complex surface-associated proteins (SAPs) in search for diagnostic biomarkers

### Ahmad Ibrahim Bagudo^1,2^, Azian Harun^1^, Kirnpal Kaur Banga Singh^1^

#### ^1^Department of Medical Microbiology and Parasitology, School of Medical Sciences, Health Campus, Universiti Sains Malaysia, Malaysia; ^2^Department of Microbiology, Faculty of Life Sciences, Kebbi State University of Science and Technology, Nigeria

##### **Correspondence:** Kirnpal Kaur Banga Singh (kiren@usm.my)

From the International Conference on Molecular Diagnostics & Biomarker Discovery 2020 (MDBD2020)

Kota Bharu, Malaysia. 17 – 18 November 2020

**Background**

In the clinical setting, *Acinetobacter baumannii* (ACB) complex is composed of three phenotypically indistinguishable Acinetobacter species (*A. baumannii, A. nosocomialis, and A. pittii*). Culture methods and automated techniques remain the gold standards for the clinical identification of ACB complex with satisfactory results. However, none of the two approaches can provide results within 24 hours. Serological tests have the potential to be used as an alternative diagnostic option for the rapid detection of ACB complex. Therefore, exploring potential diagnostic antigen candidates among the SAPs of the ACB complex will pave the way for the future development of serological tests.

**Methodology**

SAPs of Acinetobacter were extracted, followed by SDS-PAGE profiling of the proteins. Antigenicity of the SAPs was determined by Western blotting, against sera from Acinetobacter infected patients.

**Results and Discussion**

The Western blot analysis of SAPs revealed six immune-reactive proteins of 96.7 to 32.0 kDa in sizes exclusive to A. baumannii. Further analysis with non-baumannii Acinetobacter showed two proteins (36 and 22 kDa) were exclusives to A. nosocomialis and three proteins (75 to 21 kDa) exclusive to A. pittii.

**Conclusion**

This finding unveiled the potentials of Acinetobacter SAPs as a candidate for the development of rapid diagnosis for early detection and differentiation of *Acinetobacter baumannii* complex members in the clinical settings.

## O-13 Reverse transcription loop-mediated isothermal amplification for the detection of SARS-CoV-2: a preliminary report

### Godwin Attah Obande^1^, Kirnpal Kaur Banga Singh^1^, Suharni Mohamad^2^, Zakuan Zainy Deris^1^, and Aziah Ismail^3^

#### ^1^Department of Medical Microbiology and Parasitology, School of Medical Sciences, Health Campus, Universiti Sains Malaysia, Kelantan, Malaysia; ^2^School of Dental Sciences, Health Campus, Universiti Sains Malaysia, Kubang Kerian, 16150 Kelantan, Malaysia; ^3^Institute for Research in Molecular Medicine, Health Campus, Universiti Sains Malaysia, Kelantan, Malaysia

##### **Correspondence:** Aziah Ismail (aziahismail@usm.my)

From The International Conference on Molecular Diagnostics & Biomarker Discovery 2020 (MDBD2020)

Kota Bharu, Malaysia. 17 – 18 November 2020

**Background**

SARS-CoV-2 infection was first reported in the Hubei Province of Wuhan, China [1]. The viral agent has rapidly spread to every part of the world via rapid person-to-person by both symptomatic and asymptomatic individualss through droplets, aerosols and fomites [2]. The current real-time RT-PCR workflow is cumbersome, time-consuming, expensive and requires trained personnel for its application, thereby limiting its use in resource-limited settings which are not spared from the virus infection [3]. This research aims to develop a specific, rapid and simple assay for detection of SARS-CoV-2 using reverse transcription loop-mediated isothermal amplification (RT-LAMP).

**Methodology**

Genome sequences of nucleocapsid phosphoprotein (N) gene of SARS-CoV-2 were retrieved from the databases in the National Center for Biotechnology Information (NCBI) and the Global Initiative for Sharing All Influenza Data (GISAID) and aligned with other related coronaviruses, using the Mega-X software. Primers specific for SARS-CoV-2 were designed from conserved regions of the GenBank reference sequence NC_045512.2 using the PrimerExplorer version 5*. In silico* specificity of the primers was checked, using the NCBI BLAST and BioEdit software. Primer sets were screened for their efficiency to amplify the SARS-CoV-2 N gene using synthetic gene fragments, in vitro transcribed RNA and clinical samples confirmed by real-time PCR assay. Conventional LAMP and RT-LAMP where performed in the Loopamp real-time turbidimeter LA500 and both amplified products were detected in real-time and by agarose gel electrophoresis.

**Results and Discussion**

Six primer sets were designed and screened for the detection of SARS-Cov-2 N gene based on the alignment analysis. The findings revealed that only four primer sets were unique to SARS-CoV-2 and showed no cross-reaction with SARS-CoV. Two primer sets were able to detect SARS-CoV-2 within 20 minutes, and the other two sets within 40 minutes of incubation. Nucleotide mismatch of the primers with other related coronaviruses (human coronavirus strains 229E, NL63, HKU1, OC43, SARS and MERS) ranged from 5.5% to 57%. The *in silico* analysis showed a high likelihood of the primers discriminating between SARS-CoV-2 and other related coronaviruses that infect humans.

**Conclusion**

The designed primer sets have potential to accurately and rapidly detect N gene of SARS-CoV-2 via RT-LAMP. Being a simple assay that does not require sophisticated equipment [3], the RT-LAMP assay can be used with visual detection or lateral flow dipstick for testing of suspected cases in resource-limited settings.

**References**

[1] World Health Organization (2020a) Coronavirus disease 2019 (COVID-19) Situation Report – 79. Available at: https://www.who.int/docs/default-source/coronaviruse/situation-reports/20200408-sitrep-79-covid 19.pdf?sfvrsn=4796b143_4 (Accessed: 9 April 2020).

[2] Liu Y, et al. The reproductive number of COVID-19 is higher compared to SARS coronavirus. J. Travel Med. 2020, pp. 1–4. doi: 10.1093/jtm/taaa021.

[3] Estrela, P. F. N. et al. Ten-minute direct detection of Zika virus in serum samples by RT-LAMP. J Virol Methods. 2019, p. 113675. doi: 10.1016/j.jviromet.2019.113675.

## O-14 Expression of dominant AMPK β-subunit isoform in post-mortem human subcutaneous adipose tissue

### Norainfarahin Zainal Aznam^1,2^, Thuhairah Hasrah Abdul Rahman^2^, Ruzi Hamimi Razali^2^, Zaliha Ismail^1,2^, Siew Sheue Feng^3^, Aletza Mohd. Ismail^2^, Mansharan Kaur Chainchel Singh^1,2,3^

#### ^1^Institute of Pathology, Laboratory and Forensic Medicine (I-PPerForM), Universiti Teknologi MARA, Sungai Buloh, Selangor, Malaysia; ^2^Faculty of Medicine, Universiti Teknologi MARA, Sungai Buloh, Selangor, Malaysia; ^3^National Institute of Forensic Medicine (IPFN), Hospital Kuala Lumpur, Malaysia

##### **Correspondence:** Mansharan Kaur Chainchel Singh (mansharan@uitm.edu.my)

From The International Conference on Molecular Diagnostics & Biomarker Discovery 2020 (MDBD2020)

Kota Bharu, Malaysia. 17 – 18 November 2020

**Background**

Adenosine monophosphate (AMP)-activated protein kinase (AMPK) is a heterotrimeric complex consisting of α-catalytic subunit (α) and two regulatory subunits (β and γ); each presents in multiple isoforms, controlling energy balance at both cellular and whole-body levels. β1 is located in chromosome 12q24.1 in many populations while in Pima Indians β2 is located in 1q21.2, under the 12q24.31 type 2 diabetes linkage peak. Mutations in phosphorylation and post-translational modification sites in β1 have influence in AMPK activity levels and/or its nuclear distribution. β1 is generally expressed in all tissue whilst in muscle, β2 is predominantly expressed. Thus, we aim to determine the dominant AMPK β-subunit isoform present in human adipose tissue to study the activation of AMPK that can contribute to the risk in type 2 diabetes.

**Methodology**

This is a cross-sectional study of subcutaneous tissue collected from 40 Malaysian male post-mortem cases (20 lean; 20 obese) aged 18 to 65 years recruited from the National Institute of Forensic Medicine (IPFN), Hospital Kuala Lumpur. This study was funded by FRGS Grant (600-IRMI/FRGS 5/3 (001/2019) and approved by both the University (600-IRMI (5/1/6)) and Ministry of Health ethics committee (ID No: 46832). Total RNA was extracted with its quantity and integrity determined by bioanalyzer before reverse transcribed to cDNA. Quantitative real-time PCR was carried out using SYBR-green as a fluorophore detection. Comparative 2^-∆∆Ct^ method was used to calculate the relative expression of each target gene; the expression levels of both genes were normalized to that of RPLP0 and HPRT1. Mean differences of β1 and β2 in both lean and obese samples were determined by independent t-test for two-sample comparisons using SPSS 26.0 (SPSS Inc., USA).

**Results and Discussion**

There was no significant difference in the fold change for β1 gene (M=1.22, SD=0.69) and β2 gene, M=1.22, SD=0.77; t (38)= 0.007, p=0.279 (two-tailed) in lean samples. The magnitude of differences in the means (mean difference = 0.002, 95% CI: -0.468 to 0.471) was very small (eta squared = 0.001). Similarly there was no significant difference in fold change for β1 gene (M = 1.30, SD = 0.84) and β2 gene, M = 1.46, SD = 1.34; t (38) = -0.45, p = 0.12 (two-tailed) in obese samples. The magnitude of the differences in the means (mean difference = -0.16, 95% CI: -0.87 to 0.56) was very small (eta squared = 0.005). Although both β1 and β2 subunit isoforms consist a central carbohydrate binding molecule (CBM), the CBM of β2 appears to have a higher affinity for glycogen and glucose oligosaccharides thus explaining the β2 predominantly expressed in skeletal muscle. All the known allosteric activators that bind to the allosteric drug and metabolite (ADaM) binding site have a much higher affinity for complexes containing β1 rather than β2.

**Conclusion**

Results show that there is no dominant β-subunit isoform present in subcutaneous adipose tissue of both lean and obese samples. Therefore, future research may be warranted to provide more information on the structure and interaction of these isoforms for AMPK activation.

## O-15 Development of sandwich ELISA for detection of soluble PD-L1 (sPD-L1) in normal human serum

### Nur Amira Khairil Anwar, Elis Rosliza@Zeti Atikah Bt Mohd Adzmi and Noor Fatmawati Mokhtar

#### Institute for Research in Molecular Medicine (INFORMM), Higher Institution Centre of Excellence (HICoE), Universiti Sains Malaysia, Health Campus, 16150 Kubang Kerian, Kelantan, Malaysia

##### **Correspondence:** Noor Fatmawati Mokhtar (fatmawati@usm.my)

From The International Conference on Molecular Diagnostics & Biomarker Discovery 2020 (MDBD2020)

Kota Bharu, Malaysia. 17 – 18 November 2020

**Background**

Patient’s eligibility for anti-PD-L1 therapy requires PDL-1 tissue assessment assay using FDA-approved companion diagnostics. Tissue removal which is invasive is not applicable for monitoring treatment response. For post-treatment assessment, evaluation of various serum tumour markers are usually employed e.g. for breast cancer, CA 15-3, CA 27.29, and carcinoembryonic antigen (CEA) [1]. Currently, high levels of soluble PD-L1 (sPD-L1) in peripheral blood has been reported in many solid tumours which corresponded with high levels of tissue PD-L1 [2] and has a clinical potential as serum tumour marker for treatment response monitoring. This study was designed to develop an ELISA based assay for sPD-L1 detection in human serum sample.

**Methodology**

Five millilitres of blood from a healthy donor was collected (USM/JEPem/18120775) and allowed to clot at room temperature for 30 minutes before centrifuged at 2,500×*g* for 10 minutes. The resulting supernatant was taken out and stored at −80°C until usage. Sandwich ELISA was designed for sPD-L1 assay in which the capture and detection antibodies used were commercialised anti-PD-L1 mAbs; (28-8) (Abcam, USA) and 22C3 (Dako, USA), respectively. For optimal mAbs concentration, microtiter 96-well plate (Kirgen, USA) was coated with capture 28-8 (concentration range, 1.9-2000 ng/ml) in pH 7.4 PBS at 37°C for 1 hour. The coating solution was removed, and unoccupied binding sites were blocked with 5% non-fat dry milk in PBS-0.1% Tween-20 for another hour. Serum was added and incubated for 1 hour at 37°C before addition of detection 22C3 (concentration range, 1.9-2000 ng/ml) in pH 7.4 PBS for 1 hour at 37°C. After the removal of 22C3, anti-mouse conjugated with streptavidin-HRP (Abcam, USA) was added for 1 hour at 37°C and revealed with TMB substrate (Abcam, USA). Reaction was terminated using 2M H2SO4 and optical density was measured at 450 nm (Molecular Devices, USA).

**Results and Discussion**

ELISA is a practical assay for antigen detection and quantification. Its modification, sandwich ELISA i.e. utilises two antibodies possessing different epitopes further enhances the assay’s specificity. Our sandwich ELISA PD-L1 assay utilized two commercialised anti-PD-L1, 22C3 and 28-8 where 28-8 presented better as capture antibody than 22C3. The assay able to detect sPD-L1 in human serum at optimal concentration of both antibodies achieved at 500 ng/mL. The optimum dilution factor for serum sample was also identified, 1:50. Concordantly, detectable sPD-L1 in normal human serum has been reported elsewhere mostly using sandwich ELISA [3]. In those reports, the total protocol time for detection was >12 hours but in this study, it took only 8 hours.

**Conclusion**

A sandwich ELISA for sPD-L1 detection in human serum is developed. The next step is to evaluate the efficiency of this assay to detect sPD-L1 in breast cancer patients and investigate its suitability for monitoring response to therapies especially, anti-PD-L1 immunotherapy.

**References**

[1] Thariani R, Henry NL, Ramsey SD, Blough DK, Barlow B, Gralow JR, et al. Is a comparative clinical trial for breast cancer tumor markers to monitor disease recurrence warranted? A value of information analysis. J Comp Eff Res. 2013;2(3):325-34.

[2] Wei W, Xu B, Wang Y, Wu C, Jiang J, Wu C. Prognostic significance of circulating soluble programmed death ligand-1 in patients with solid tumors: A meta-analysis. Medicine (Baltimore). 2018;97(3):e9617.

[3] Chen Y, Wang Q, Shi B, Xu P, Hu Z, Bai L, et al. Development of a sandwich ELISA for evaluating soluble PD-L1 (CD274) in human sera of different ages as well as supernatants of PD-L1+ cell lines. Cytokine. 2011;56(2):231-8.

## O-16 PCR optimisation for detection of familial hypercholesterolaemia gene mutations in the Malaysian population

### Norhidayah Rosman^1,2^, Yung-An Chua^1^, Alyaa Al-Khateeb^1,2^, Hapizah Mohd Nawawi^1,2^ and Ang-Lim Chua^2^

#### ^1^Institute for Pathology, Laboratory and Forensic Medicine (I-PPerForM), Universiti Teknologi MARA, Sungai Buloh, Selangor, Malaysia; ^2^Faculty of Medicine, Universiti Teknologi MARA, Sungai Buloh, Selangor, Malaysia

##### **Correspondence:** Ang-Lim Chua (anglim@uitm.edu.my)

From the International Conference on Molecular Diagnostics & Biomarker Discovery 2020 (MDBD2020)

Kota Bharu, Malaysia. 17 – 18 November 2020

**Background**

Familial hypercholesterolaemia (FH) is a genetic disorder that leads to the severe elevation of low-density lipoprotein cholesterol (LDL-C) level. Early detection of FH is important to reduce the risk of premature coronary artery disease (pCAD). Currently, next-generation sequencing (NGS) is used for molecular confirmation of FH patients worldwide. However, this method is time-consuming and expensive. Thus, we have optimised a PCR-based FH molecular method to detect FH gene mutations, targeting the known low-density lipoprotein receptor *(LDLR)* and apolipoprotein B (*APOB)* pathogenic variants in Malaysia.

**Methodology**

Optimisation of the PCR method, determination of limit of detection (LoD) and preliminary evaluation were performed. This study includes 20 unique primers sets of wild-type (WT) and pathogenic (MUT) *LDLR* and *APOB* gene variants. The PCR was run in a 20μL PCR master mix and visualised on 2% agarose gel. PCR parameters such as annealing temperature, annealing time, and extension time also the number of cycles were optimised in this study. The LoD of positive control (oligo-synthesised) was tested using 10x serial dilutions with concentration 1.0x10^0^ to 10^-9^ ng/μL. In contrast, the LoD of patients’ DNA was tested using 5x serial dilutions with concentration ranging from 10.0x10^0^ to10^-4^ ng/μL. Each patients’ DNA was analysed in five replicates. The LoD was defined as the lowest concentration of at least one positive replicate.

**Results and Discussion**

The optimal annealing temperature was determined in the range of 60 to 63ºC, with annealing times in the range of 35-40s. The optimum extension times were in the range of 40-50s. The thermocycling was run for 28 to 30 cycles to get the best results. Maintaining an optimal range for each protocol parameter is essential to provide a margin error for the potential protocol users and reduce non-specific amplification [1]. The LoD was performed after the optimisation of the PCR method parameters. The LoD for the positive control and positive FH patients’ DNA was 1.0X10^-9^ ng/μL and 10.0X10^-1^ ng/μL, respectively. A positive band was detected at lower limits with the positive control DNA than patients’ DNA, as it was a pure synthetic target [2]. Replicates were used in this study to reduce false-negative for both positive control and the patients’ DNA.

**Conclusion**

The PCR assays for 20 gene variants have been optimised to run at the same PCR parameters, and the assay has an LoD for patients’ DNA of 10.0X10^-1^ ng/μL. Optimising PCR conditions to work in a range for each thermocycling parameter is crucial to ensure that the method is robust and applicable to different users with heterogeneous laboratory settings.

**References**

[1] D. Zhang, Y. Li, X. Zhang, Y. Cheng, and Z. Li, “Enhancement of the polymerase chain reaction by tungsten disulfide,” *RSC Adv.*, vol. 9, no. 17, pp. 9373–9378, Aug. 2019, doi: 10.1039/C8RA09689A.

[2] I. Fahoum *et al.*, “Characterization of Factors Affecting the Detection Limit of EGFR p.T790M in Circulating Tumor DNA,” *Technol. Cancer Res. Treat.*, vol. 17, Aug. 2018, doi: 10.1177/1533033818793653.

## O-17 Affimer: A versatile affinity reagent for biosensor and bioanalytical applications for colorectal cancer biomarker detection

### Shazana Hilda Shamsuddin^1,2^, Timothy D. Gibson^2^, Darren C. Tomlinson^3^, Michael J. McPherson^3^, David G. Jayne^4^, Paul A. Millner^2^

#### ^1^Department of Pathology, School of Medical Sciences, Health Campus, Universiti Sains Malaysia, 16150, Kelantan, Malaysia; ^2^The Leeds Bionanotechnology Group, School of Biomedical Sciences, University of Leeds, LS2 9JT, Leeds, United Kingdom; ^3^Astbury Centre for Structural and Molecular Biology, University of Leeds, LS2 9JT, Leeds, United Kingdom; ^4^Leeds Institute of Medical Research, University of Leeds, LS9 7TF, Leeds, United Kingdom

##### **Correspondence:** Shazana Hilda Shamsuddin (shazana.hilda@usm.my); Paul A. Millner (P.A.Millner@leeds.ac.uk)

From The International Conference on Molecular Diagnostics & Biomarker Discovery 2020 (MDBD2020)

Kota Bharu, Malaysia. 17 – 18 November 2020

**Background**

Morbidity and mortality rates of colorectal cancer (CRC) remain among the highest in cancer cases due to late detection. Conventional diagnostics are complicated, often invasive and time-consuming with variable sensitivity and specificity. Most CRC diagnostic tools are antibody-based which suffer from shortcomings including stability and batch variability issues, expensive and complicated for mass production. Hence, development a highly sensitive, specific and rapid diagnosis device using non-antibody based, such as a biosensors is needed. The main aim of this study was to evaluate the potential of Affimer protein (12.6 kDa) as a novel alternative affinity binding reagent for carcinoembryonic antigen (CEA) detection and its application in bioanalytical and biosensor assays. CEA, is the only validated blood biomarker routinely used for prognostic screening of advanced CRC.

**Methodology**

Anti-CEA Affimers were screened and isolated using phage display libraries. Characterisation of the specificity and selectivity binding of Affimers against CEA were evaluated using affinity-fluorescence staining on LoVo cells, affinity-precipitation assay and SPR analysis for the determination of kinetic binding. The optimized CEA binders were fabricated on DropSens screen printed gold electrodes coated with a novel non-conducting polymer layer, polyoctopamine (POct), for CEA detection. Electrochemical characterisation of Affimer-based biosensors was performed via cyclic voltammetry, electrochemical impedance spectroscopy and protein blotting.

**Results and Discussion**

The Affimer binders contain two variable peptide regions that form the binding site for CEA. Validation assays showing Affimers isolated from the phage display library screening, have high-affinity binding (within nM range) and can bind specifically and selectively to protein epitopes of CEA from cell culture lysate and on fixed cells. They recognise multiple forms of functional CEA including the fully glycosylated native form as well as incompletely glycosylated and deglycosylated forms. It is important to highlight that fluorescent staining using anti-CEA Affimer as a monoclonal reagent offers additional advantages and cost effectiveness as it does not require secondary antibody for detection which sometimes can cross reacts and introduces false positive staining. Inclusion of single cysteine residue at the C-terminus of the Affimer facilitated oriented immobilization of the bioreceptor during the fabrication of biosensors. A highly sensitive and specific Affimer-based impedimetric biosensor was successfully developed for the detection of CEA with a limit of detection of 1 fM, which is significantly lower than the basal clinical levels of 25 pM, and a linear detection range of 1 – 100 fM. This label-free biosensor offers simple fabrication, rapid response time (5 min) and requires small-sample volume (10 μl).

**Conclusion**

Therefore, findings from this study have demonstrated that Affimers could be an attractive alternative to antibody in bioanalytical application. Affimers have the potential to be utilised as molecular probes in diagnostic imaging of CEA-expressing tumours, targeted therapy and purification of CEA protein using the affinity separation assay. The proposed Affimer-based impedimetric biosensor has strong potential for the development of point-of-care (POC) device for CEA detection notably in colorectal cancer.

## O-18 Whole mitochondrial genome sequencing in cardiomyopathy patients in Malaysia

### Kuan Sheh Wen^1^, Tan E-Wei^1^, Alexander Loch^2^, Tan Lay Koon^3^, Chua Kek Heng^1^ and Kee Boon Pin^1*^

#### ^1^Department of Biomedical Science, Faculty of Medicine, Universiti Malaya, Kuala Lumpur, Malaysia; ^2^Department of Medicine, Faculty of Medicine, Universiti Malaya, Kuala Lumpur, Malaysia; ^3^National Heart Institute, Kuala Lumpur, Malaysia

##### **Correspondence:** Kee Boon Pin (bpkee@um.edu.my)

From The International Conference on Molecular Diagnostics & Biomarker Discovery 2020 (MDBD2020)

Kota Bharu, Malaysia. 17 – 18 November 2020

**Background**

Cardiomyopathy (CMP) is commonly known to affect the heart muscle and its ability to pump blood around the body. Signs and symptoms such as shortness of breath after exertion and fatigue are normally presented. CMP is currently without cure. The two most common subtypes of CMP are hypertrophic cardiomyopathy (HCM) and dilated cardiomyopathy (DCM). CMP is expected to affect 1 in every 2,500 persons. The exact etiology of CMP remains unknown. However, studies suggest environmental and genetic factors play a vital role in the onset of CMP. About half of the CMP cases are familial, reflecting the predisposing role of genetic composition on the development of CMP. Mutations in genes encoding sarcomere, cytoskeletal and desmosome were found to be related to CMP. Besides, studies have shown that mitochondrial dysfunction was also one of the causes of CMP. The mutations in mitochondrial DNA (mtDNA) were expected to have direct impact on the energy production in cardiomyocyte, thus leading to the development of CMP. Hence, in this study, we aimed to investigate the full mutation spectrum of mitochondrial genome in Malaysian CMP patients.

**Methodology**

A total of 146 CMP patients were recruited in this study. Demographic and clinical data were retrieved from hospital database. Genomic DNA was extracted from venous blood using conventional phenol-chloroform extraction method. DNA quality and quantity were assessed using nanophotometer. Long-range PCR was employed to generate amplicons for library preparation for Next-Generation Sequencing (NGS) run in Miseq instrument. The sequences generated were analyzed and mitochondrial variants were called. In-silico software were used to predict the effect of mutation and haplogroup was assigned for each subject. Statistical analyses were computed.

**Results and Discussion**

Among these 146 patients, 99 (67.8%) were male and 47 (32.2%) were female, aged 19 to 82 years old, with an average of 50.7±15.4 years old. Fifty-seven (39.0%) were HCM patients while 89 (61.0%) were DCM patients. The patients comprised of different ethnicity groups: 67 (45.9%) Malay, 55 (37.7%) Chinese, 17 (11.6%) Indian and 7 (4.8%) others. The overall average coverage of whole mitochondrial genome sequencing was 1,137.2±702.7×. The sequencing revealed 31 pathogenic variants: 21 were non-synonymous, one was start-loss, seven were in *MT-tRNA* and two were in *MT-rRNA*. The non-synonymous variants included six in *MT-ND1*, three in *MT-CYB* and *MT-ND4* respectively, two in *MT-CO3, MT-ND3, MT-ND5* and *MT-ND6* respectively and one in *MT-CO1*. The stop-loss variant was observed in *MT-ND5*. Besides, the two variants observed in *MT-rRNA* were both in *MT-RNR1*. The variants observed in *MT-tRNA* were: two in *MT-TL1* and *MT-TL2* respectively and one in *MT-TC*, *MT-TF* and *MT-TY* respectively. Five of the 31 variants were previously reported with confirmed pathogenicity: m.1555A>G, m.3697G>A, m.10197G>A, m.11778G>A and m.12315G>A. Noteworthy, six pathogenic variants were observed in *MT-ND1*. *MT-ND1* is responsible to encode Complex I subunit in electron transport chain. Mutation in this gene causes production of aberrant protein and may lead to disruption of oxidative phosphorylation. This causes a ramp down of energy production, leaving the energy demand of cardiomyocyte unfulfilled, thus causing CMP.

**Conclusion**

In conclusion, the whole mitochondrial genome sequencing revealed 31 pathogenic variants in CMP patients in Malaysia. The pathogenic variants may be involved in the development of CMP. Further analyses are required to elucidate the role of these pathogenic variants in the pathogenesis of CMP.

## P-2 Development of an in-house real-time RT-PCR assay for detection of novel coronavirus (SARS-CoV-2)

### Zarina Mohd Zawawi, Jeyanthi Suppiah, Tengku Rogayah Tengku Abdul Rashid, Mohamad Helmi Jalaludin, Manisya Zauri Ab Wahid, Muhammad Afif Azizan, Rozainanee Mohd Zain, Ravindran Thayan

#### Virology Unit, Infectious Disease Research Centre, Institute for Medical Research, National Institutes of Health Complex, Ministry of Health Malaysia, Selangor, Malaysia

##### **Correspondence:** Zarina Mohd Zawawi (zarina.zawawi@moh.gov.my)

From The International Conference on Molecular Diagnostics & Biomarker Discovery 2020 (MDBD2020)

Kota Bharu, Malaysia. 17 – 18 November 2020

**Background**

The coronavirus disease (COVID-19) pandemic caused by severe acute respiratory syndrome coronavirus 2 (SARS-CoV-2) has affected almost every country globally. The first reports of COVID-19 by China to the World Health Organization (WHO) highlights the critical need for developing a molecular assay as part of the outbreak preparedness. The aim of this study was to develop a real time assay for early detection of SARS-CoV-2 virus in clinical samples.

**Methodology**

Primers and probe sequences targeting the conserved region of the envelope gene of SARSCoV-2 as well as SARS-like CoV’s were designed. Real-time RT-PCR assay was optimized and performed in a CFX-96 thermal cycler. Viral nucleic acid was extracted from clinical samples; sputum, nasal swab, throat swab and nasopharyngeal and oropharyngeal swabs. The in-house assay was then evaluated by testing a total of 583 suspected cases concurrently with RdRp WHO protocol.

**Results and Discussion**

Primers and probe (WH nCoV-F, WH nCoV -R and WH nCoV-P) for SARS-CoV-2 detection were successfully developed. A total of 583 suspected cases were screened using the in-house assay. Of these 22 were confirmed positive for COVID-19. The specificity of detection was 100% and the in-house assay showed concordance with test results performed by RdRp WHO protocol. Positive amplification was observed in FAM channel and no cross reactivity was found when tested with respiratory viruses such as adenovirus and Pandemic A(H1N1)09 from archived samples in our laboratory.

**Conclusion**

The quick identification of first three cases in Malaysia was possible due to the availability of the in-house TaqMan Real-Time RT-PCR assay for detection of COVID-19. Therefore, the development of the in-house molecular assay for SARS-CoV-2 infection allows a rapid diagnosis among the suspected case in Malaysia. The developed assay was advantageous in screening and detecting SARS-CoV-2 from initial batches of specimens sent to the laboratory prior to the release of the protocol recommended by the WHO.

## P-5 Potential role of miR-125b-5p and *RASGRF2* in human multiple myeloma

### Ivyna Bong Pau Ni^1^, Ng Ching Ching^2^, Norodiyah Othman^1^, Aliza Mohd Yacob^1^ and Ezalia Esa^1^

#### ^1^Haematology Unit, Cancer Research Centre, Institute for Medical Research, National Institutes of Health, Ministry of Health, Malaysia; ^2^Faculty of Science, Institute of Biological Sciences, University of Malaya, Kuala Lumpur, Malaysia

##### **Correspondence:** Ivyna Bong Pau Ni (ivyna@moh.gov.my)

From The International Conference on Molecular Diagnostics & Biomarker Discovery 2020 (MDBD2020)

Kota Bharu, Malaysia. 17 – 18 November 2020

**Background**

Multiple myeloma (MM) is a cancer of plasma cell. Genetic and epigenetic abnormalities are thought to be contributed in the oncogenesis of MM [1]. This study aims to evaluate the role of miR-125b-5p and its biological relevance with *RASGRF2* gene in MM cells.

**Methodology**

Relative expression of miR-125b-5p and *RASGRF2* was evaluated in 12-15 MM patients, 8 cell lines and 4 normal controls by quantitative real-time PCR (qRT-PCR). RNU6 and *GAPDH* was used as internal control for miRNA and gene expression assay, respectively. 300-500nM of miR-125b-5p mimics/ inhibitors/ controls were nucleofected into 2.0 x 10^6^ RPMI-8226 or KMS-28BM MM cells. Total RNAs were extracted from the cells at 24 h/ 48 h post-transfection. The effects of miRNA mimics/ inhibitors vs controls on miR-125b-5p and *RASGRF2* expression was measured by qRT-PCR. Relative quantification of miR-125b-5p mimics/ inhibitors/ *RASGRF2* was determined using 2^-∆∆Ct^ method.

**Results and Discussion**

Aberrant expression of miR-125b-5p is found in various cancers and it functions in controlling cell proliferation, differentiation, metabolism, apoptosis, drug resistance and tumour immunity [2]. The present findings found that miR-125b-5p was differentially expressed in 8/12 MM patients (5/12 up-regulated & 3/12 down-regulated) and 8/8 MM cell lines (4/8 up-regulated & 4/8 down-regulated). Interestingly, a gene involved in RAS related pathway: *RASGRF2* was under-expressed in 15/15 MM patients and 8/8 MM cell lines. Sequence matching information from TargetScan database shows that there is a possible correlation between miR-125b-5p and *RASGRF2*. This indicates that miR-125b-5p may interact with *RASGRF2* in myelomagenesis. By using RPMI-8226 as a model, miR-125b-5p mimics was successfully delivered into the cells and increased miR-125b-5p expression by 27-folds. On the other hand, miR-125b-5p inhibitors had successfully down-regulated miR-125b-5p by 95% in KMS-28BM. However, the qRT-PCR results revealed that miR-125b-5p mimics and inhibitors did not alter *RASGRF2* expression in RPMI-8226 and KMS-28BM suggesting that miR-125b-5p do not modulate *RASGRF2* at transcriptional level.

**Conclusion**

The preliminary findings of this study show that miR-125b-5p expression is successfully induced and reduced by miR-125b-5p mimics and inhibitors, respectively in RPMI-8226 and KMS-28BM. Changes in miR-125b-5p expression do not modulate *RASGRF2* at transcriptional level in these cells. Further experimental investigations are needed to determine whether miR-125b-5p mediate translational repression of RASGRF2 in MM and the roles of miR-125b-5p in proliferation and apoptosis of myeloma cells.

**References**

[1] Koura DT, et al. Inherited predisposition to multiple myeloma. Ther. Adv. Hematol. 2013, 4(4): 291-297.

[2] Wang Y, et al. The emerging roles of miR-125b in cancers. Cancer Manag. Res. 2020, 12:1079-1088.

## P-6 Genetic variation of childhood acute lymphoblastic leukaemia by next-generation sequencing (NGS): a case report

### Norodiyah Othman^1,2^, Ivyna Bong Pau Ni^1^, Fazlin Mohd Fauzi^**1**^ and Ezalia Esa^1^

#### ^1^Haematology Unit, Cancer Research Centre, Institute for Medical Research, National Institutes of Health Complex, Setia Alam. 40170, Shah Alam, Selangor, Malaysia; ^2^Faculty of Pharmacy, Universiti Teknologi MARA, Selangor Branch, Puncak Alam Campus, 42300 Bandar Puncak Alam, Selangor, Malaysia

##### **Correspondence:** Norodiyah Othman (norodiyah@moh.gov.my)

From The International Conference on Molecular Diagnostics & Biomarker Discovery 2020 (MDBD2020)

Kota Bharu, Malaysia. 17 – 18 November 2020

**Background**

Acute Lymphoblastic Leukaemia (ALL) with the *ETV6/RUNX1* fusion gene is the most common malignant disorder found in children. Although the chromosomal abnormality was the benchmark for ALL development, the identification of the genetic changes that co-exist with the fusion gene is necessary for both prognosis and disease treatment [1]. Therefore, we report genetic variation present in one case of four years old female children with a history of precursor B-ALL carrying the *ETV6/RUNX1* fusion gene using whole-exome sequencing.

**Methodology**

DNA was isolated from bone marrow samples using the QIAmp DNA Blood Mini kit and subsequently checked for DNA integrity and quality using gel electrophoresis and Qubit 2.0 fluorometer. Genomic DNA was enriched using Truseq® Exome Enrichment Kit and sequenced using the Illumina MiSeq system. Read files (FASTQ) was generated from the sequencing platform via Illumina’s CASAVA pipeline version 1.8.2. Sequence alignment and variant calling were performed using CLC Genomic Workbench version 7.5 and data were further analysed using ENLIS software. Results validation was done using sanger sequencing.

**Results and Discussion**

After sequencing, the samples generated an average of 18.6 million paired reads with 99% matched to the reference genome. We detected 17,601 variations in the sample, against the human reference genome UCSC NCBI137/ hg19. Single nucleotide polymorphisms (SNPs) contributed 88% of total variations in the coding region, whereas substitution/insertion or deletion contributed about 2.1% of the variations. When taking into account the coding region and protein impact, 45% (7,993 variants) were synonymous SNPs and 40% (7,058 variants) were non-synonymous SNPs. Among a total of non-synonymous SNPs; 7,595 were missense, 65 nonsense, 26 nonstop, 12 misstart, 121 frameshifts, 86 inframe ins/del and 1,703 disrupting splices site. Using more stringent filtering, we identified 28 variants located in the coding region of 20 leukaemia-related genes. Five genes (ABL1, ARHGAP26, FLT3, NSD1, and PML) have more than one variation. Some of these variants were predicted to alter the amino acid composition of the resulting protein, using either Polyphen or SIFT prediction tools.

**Conclusion**

Detection and identification of genetic variations in leukaemia samples are important to improve the understanding of the molecular mechanisms underlying the disease progression. It also can facilitate the discovery of potential drug targets which can lead to a rationally designed treatment regimen.

**Reference**

[1] Ilyas AM, et al. Next generation sequencing of acute myeloid leukemia: influencing prognosis. *BMC Genomics.* 2015; 16 Suppl 1; S5.

## P-7 Nisin ZP modulates MG63 osteosarcoma cell viability through apoptosis in a 3-dimensional cell culture system

### Alyaa Alkhateeb^1,2^, sharaniza AB Rahim^1^, Zulaika Roslan^3^, Muhammad Fairuz Azmi^1^, Gabriele Ruth Anisah Fromming^4^

#### ^1^Faculty of Medicine, Universiti Teknologi MARA (UiTM), Sungai Buloh, Malaysia; ^2^Institute of Pathology, Laboratory and Forensic Medicine (I-PPerForM), Universiti Teknologi MARA, Sungai Buloh, Selangor, Malaysia; ^3^Institute of Medical Molecular Biotechnology (IMMB), Faculty of Medicine, Universiti Teknologi MARA; ^4^Faculty of Medicine and Allied Health Sciences, University Malaysia Sarawak, 94300 Kota Samarahan, Sarawak

##### **Correspondence:** Alyaa Alkhateeb (alyaa@uitm.edu.my)

From The International Conference on Molecular Diagnostics & Biomarker Discovery 2020 (MDBD2020)

Kota Bharu, Malaysia. 17 – 18 November 2020

**Background**

Nisin, a bacteriocin, has been widely used as an antibiotic. Recently its anticancer properties have come under investigation especially in head and neck carcinoma. However, information on its effect on osteosarcoma cells is still lacking.

**Methodology**

The aim of this study is to determine the apoptotic effect of nisin ZP on osteosarcoma cells. MG63 osteosarcoma cells were seeded at a density of 1.5 x 104 cells/well in a 96 well V-cell G-plates with 100 μl of DMEM/F12 supplemented with 5% FBS culture media. The cells were incubated at 37°C and 5% CO2 overnight prior treatment with increasing concentration of nisin ZP from 100 – 600 μg/ml and further incubated. The apoptosis assay was carried out at 24 hrs and 48 hrs post-treatment.

**Results and Discussion**

The results showed that nisin ZP caused a dose-dependent apoptosis in MG63 cells, both at 24 hrs and 48 hrs post-treatment. However, the level of apoptosis was starting to decrease at 600 μg/ml in 24 hrs post-treatment indicating that the cells could be reaching the saturated concentration of nisin cytotoxicity effect. At 48 hrs post-treatment, the apoptosis process was affected but at a lower rate. This could indicate that the cells were undergoing a recovery or resistance process after 48 hrs post-treatment. It is concluded from this study that nisin ZP affect osteosarcoma cell viability through apoptosis thus it has a potential to be used as therapeutic agent for osteosarcoma treatment.

**Conclusion**

Nisin has an apoptotic effect on osteosarcoma cells in a 3-dimensional cell culture system.

**References**

[1] Markus Rimanna, Sandra Laternser , Ana Gvozdenovic , Roman Muff , Bruno Fuchs , Jens M. Kelmc, Ursula Graf-Hausner. An in vitro osteosarcoma 3D microtissue model for drug development. Journal of Biotechnology 189 (2014) 129–135.

[2] Alexander B. Mohseny, Pancras C. W. Hogendoorn, and Anne-Marie Cleton-Jansen. Osteosarcoma Models: From Cell Lines to Zebrafish. Sarcoma Volume 2012, Article ID 417271, 11

## P-8 Isolation by culture and polymerase chain reaction identification of LipL21 gene of pathogenic *Leptospira* sp. from cockroach body surface samples in Chow Kit Market, Kuala Lumpur

### Latifah I^1^, Abdul Halim A^1^, MY Aliza^1^, H Roslina^2^, Edmond Zulkiflil^1^, Nurul Annissha AS^3^, Asmah H^3^ and Shafariatul Akmar I^3^

#### ^1^Institute for Medical Research, Jalan Pahang, 50588, Kuala Lumpur, Malaysia; ^2^Institute for Clinical Research National, Institutes of Health, 40170, Setia Alam, Selangor, Malaysia; ^3^Universiti Kebangsaan Malaysia, Jalan Raja Muda Abdul Aziz, 50400, Kuala Lumpur, Malaysia

##### **Correspondence:** Latifah I (latifah.ibrahim@moh.gov.my)

From The International Conference on Molecular Diagnostics & Biomarker Discovery 2020 (MDBD2020)

Kota Bharu, Malaysia. 17 – 18 November 2020

**Background**

Cockroaches interact more or equal with humans compared to rats. Situation of infected rats contaminating their habitats with its urine and sharing the same habitats with cockroaches in the urban area might possibly contaminate the body and the digestive tract of the cockroaches with *Leptospira* sp. Therefore this study has attempted to demonstrate the potential role of cockroaches as carriers of pathogenic *Leptospira* sp.

**Methodology**

A total of 135 swab samples from body surface were collected from trapped cockroaches. These samples were cultured in EMJH enriched media and 15 of these samples (11.1 %) were found to be positive when observed under 40x dark field microscope. Genomic DNA was extracted from all the 15 native isolates for PCR. PCR products were also sequenced and BLAST analysis was performed on each positive isolate.

**Results and Discussion**

All the 15 isolates generated the expected 561 base pair band with PCR when a set of primers known to amplify LipL21 gene was utilized. These results showed that the primers were suitable to be used for the identification of pathogenic leptospira from swab sample of body surface of the cockroaches. BLAST analysis performed on each sequence of the isolates confirmed the pathogenic status of all these native isolates and LipL21 gene was detected in all the Leptospira isolates.

**Conclusion**

The research proves that the cockroach is able to act as a transporter of pathogenic *Leptospira* sp. and long-term epidemiology research has to be carried out to verify that the cockroach is the true source of infection towards the human.

**References**

[1] Fatin Nadia. Pengenalpastian Leptospira sp. di kalangan populasi roden daripada beberapa kawasan terpilih di Kuala Lumpur, Malaysia. Tesis Universiti Kebangsaan Malaysia. 2016.

[2] Gonzalez-Astudillo, V, et al. Synanthropic cockroaches (Blattidae: Periplaneta sp) harbor pathogenic Leptospira in Colombia. J Med Entomol. 2015. 53(1):177-182.

[3] Latifah 1 et al. Isolation by culture and PCR identification of LipL32 gene of pathogenic Leptospira sp. in wild rats of Kuala Lumpur. Malay J Pathol. 2017. 39(2):161-166.

## P-9 Successful isolation and propagation of Malaysian SARS-CoV-2 Strain in Vero E6 cell lines

### Siti Nur Zawani Rosli, Mohammad Ridhuan Mohd Ali, Sitti Rahmawati Dimeng, Nur Afrina Muhamad Hendri, Hana Farizah Zamri, Jeyanthi Suppiah, Zarina Mohd. Zawawi, Jama’ayah Mohamed Zahidi, Nur Asyura Nor Amdan, Rozainanee Mohd Zain, Ravindran Thayan and Norazah Ahmad

#### Institute for Medical Research (IMR), National Institutes of Health (NIH), Setia Alam, Malaysia

##### **Correspondence:** Siti Nur Zawani Rosli (sn.zawani@moh.gov.my)

From The International Conference on Molecular Diagnostics & Biomarker Discovery 2020 (MDBD2020)

Kota Bharu, Malaysia. 17 – 18 November 2020

**Background**

The emergence of a novel coronavirus in Wuhan, Hubei Province, China in December 2019 has posed a global challenge for the public health worldwide. This virus was identified as the Severe Acute Respiratory Syndrome- Coronavirus-2 (SARS-CoV-2) and are diverse into 3 main variants; A, B and C. There are many studies carried out on COVID-19 focusing on detection, therapies, and others. In this present study, we have succeeded in isolating and propagating SARS-CoV-2 from clinical specimens obtained from the surveillance testing of COVID-19 cases.

**Methodology**

The clinical samples were inoculated in the Vero E6 cell line. Cytopathic effect (CPE) was observed in the following days and virus cultures was harvested when CPE was at around 70-80% (3 d.p.i). Subsequently, viral copy numbers were determined by real-time RT-PCR.

**Results and Discussion**

The virus isolated were characterized and shown to be from ancestral type B. Isolation and propagation of the pathogens from the patients is one of the important steps in order to understand the virus better.

**Conclusion**

The ability to propagate the virus will then provide the capacity for further research into tackling the spread of COVID-19.

## P-10 Serotonergic modulation within the neurogenic niche of the postnatal spinal cord

### Nurhafizah Ghani^1^, Nazlahshaniza Shafin^2^, Susan A.Deuchars^3^, Jim Deuchars^3^

#### ^1^School of Dental Sciences, Universiti Sains Malaysia, Malaysia; ^2^School of Medical Sciences, Universiti Sains Malaysia, Malaysia; ^3^School of Biomedical Sciences, University of Leeds, UK

##### **Correspondence:** Nazlahshaniza Shafin (drshaniza@usm.my)

From The International Conference on Molecular Diagnostics & Biomarker Discovery 2020 (MDBD2020)

Kota Bharu, Malaysia. 17 – 18 November 2020

**Background**

Neurogenesis is the differentiation of neural stem cells into neurones, astrocytes and oligodendrocytes. There is evidence for postnatal neurogenesis in the central canal area of the spinal cord [1, 2].

**Methodology**

To screen whether activation of specific 5-HT receptors may influence the activity ependymal cells of the spinal cord and to determine the location of 5-HTergic fibres, patch-clamp and immune fluorescence methods was performed. To investigate the relationship between neurogenic niches and 5-HTergic fibres, 5-HT immunofluorescent staining was done on nestin-GFP (green fluorescent) spinal cord sections. To determine the effect of endogenous serotonin on cell proliferation of spinal cord, C57BI/6 mice (6-8 week, n=4) were injected with fluoxetine hydrochloride (Flx) in 10 mg/kg for 10 days and the thymidine analogue 5-ethynyl-2’-deoxyuridine (EdU) in 10 mM was injected for the last 5 days to determine the extent of cell proliferation. The control group C57BI/6 (6-8 week, n=4) were injected with saline and EdU for 10 days and the last 5 days respectively.

**Results and Discussion**

5-HT immunofluorescent terminals observed in closed apposition to ependymal cells. 5-HTergic fibres were found in the intermediolateral cell column (IML) region of grey matter and the fibres travelled medially from IML to the central region and surrounded the central canal. Whole-cell patchclamp recordings revealed that 5-HT (10 μM) caused opposing effects consisting of depolarisation or a hyperpolarisation in different ependymal cells. Administration of the 5-HT reuptake inhibitor, Flx promoted greater numbers of proliferating cells in the dentate gyrus (DG) of the hippocampus (used as a positive control), grey matter in the thoracolumbar spinal cord. However, there were no significant differences in the numbers of EdU+ve cells between Flx-treated and control mice in either the central canal or white matter. Furthermore, the proportion of new cells that became astrocytes, oligodendrocytes and neurones were not significant too.

**Conclusion**

A population of ependymal cells can respond to 5-HT with both depolarisations and hyperpolarisations. Blockade of 5-HT receptor mediated depolarisations with cinanserin and hyperpolarisations with 8-OHDPAT showed the presence of 5-HT2 and 5-HT1A receptors in spinal cord. Flx increases level of 5-HT by blocking re-uptake of 5-HT to the presynaptic terminals. This endogenous 5-HT promoting high levels of proliferating cell in spinal cord since 5-HTergic fibres present at the central canal indicate that 5-HT can influence ependymal cell and suggesting that 5-HTergic signalling may modulate the stem cells area and can influence neurogenesis.

**References**

[1] Alfaro‐Cervello, C. et al., 2012. Biciliated ependymal cell proliferation contributes to spinal cord growth. Journal of Comparative Neurology. 520(15).

[2] Barnabé-Heider, F. et al., 2010. Origin of new glial cells in intact and injured adult spinal cord. Cell stem cell. 7(4).

## P-11 Cloning, expression and purification of metallo-β-lactamase from *Glaciozyma antarctica*

### Nik Yusnoraini Yusof^1^, Abdul Munir Abdul Murad^2^, Farah Diba Abu Bakar^2^ and Mohd Firdaus-Raih^2^

#### ^1^Institute for Research in Molecular Medicine, Health Campus, Universiti Sains Malaysia, Kelantan, Malaysia; ^2^School of Bioscience and Biotechnology, Faculty of Science and Technology, Universiti Kebangsaan Malaysia

##### **Correspondence:** Nik Yusnoraini Yusof (nikyus@usm.my)

From The International Conference on Molecular Diagnostics & Biomarker Discovery 2020 (MDBD2020)

Penang, Malaysia. 17 – 18 November 2020

**Background**

Genes encoding lactamases are also abundant in various fungal families parallel to the existence of bacteria. However, the role of these genes in fungi is relatively unknown [1]. It indicates that many of these enzymes work in degradation and xenobiotic compounds as with bacteria [2]. To date, there have been no reports on the metallo-β-lactamase from psychrophilic yeast. This preliminary research is the founding model for the study of hydrolytic lactamases in fungi. In the present study, we have isolated gene sequence of metallo-β-lactamase produced by an Antarctic yeast, *G.antarctica* PI12. This study aims to amplify, clone and express metalo-β-lactamase from *Glaciozyma antactica* (GaBML).

**Methodology**

*Glaciozyma antarctica* was obtained from the School of Biosciences and Biotechnology, Universiti Kebangsaan Malaysia, Malaysia. The cDNA, encoding GaBML was isolated and amplified using reverse transcriptase-PCR (RT-PCR). The forward and reverse primers used to amplify the cDNA were 5’-ATCATATCAAGATGGCGACGAG-3’ and 5’-CGAGAGCCCTACGACCAAAA-3’, respectively. The amplified fragment was inserted into a cloning vector, transformed into *Escherichia coli* DH5 cells and plated onto a Luria–Bertani (LB) agar plate containing ampicillin (100 μg/ml). The inserted sequence was verified as metallo-β-lactamase by sequencing. The gene was then subcloned into the pET-30 Ek/LIC vector (Novagen). The generated plasmid was sequenced to assure conformity and subsequently transformed into *E. coli* BL21 (DE3) cells (Novagen). The starter culture of the positive GaBML transformant was grown in 500 mL LB broth supplemented with 50 μg/mL kanamycin in 37 °C with shaking at 200 rpm to an OD600 of ~0.5–0.6. Then, the cultures were incubated at 16 °C for 18 h with shaking at 200 rpm after induced by adding 0.1 mM isopropyl-β-D-thiogalactosidase (IPTG). The harvested cells by centrifugation at 4000 × g for 20 min were resuspended in 20 mM Tris-HCl (pH 8), 150 mM NaCl and disrupted by sonication. After sonication, the cells were centrifuged at 10000 × g for 20 min. The recombinant GaMBL protein was then purified from the supernatant via nickel-NTA affinity chromatography. The binding buffer comprised of 50 mM Tris-HCl and 150 mM sodium chloride at pH 8.0, whereas the elution buffer comprised of 50 mM Tris-HCl, 150 mM sodium chloride and 500 mM imidazole at pH 8.0. The sample purity and molecular weight were evaluated by denaturing polyacrylamide gel electrophoresis and Western blot analysis [3].

**Results and Discussion**

An open reading frame of 924 bp GaMBL cDNA was amplified and sequenced. The predicted protein consists of 307 amino acids with an estimated molecular weight of 34 kDa. The recombinant protein, GaMBL was expressed as a histidine tagged protein in *E. coli* and purified under denaturing conditions using Ni-NTA agarose. SDS-PAGE analysis of the purified GaMBL showed a single 34 kDa protein band corresponding to the expected molecular weight, including the size of the histidine tagged protein.

**Conclusion**

The metallo-β-lactamase gene from *G. antarctica* has been successfully cloned, expressed and purified. Further characterization experiments are expected to confirm the function of this novel metallo-β-lactamase from a psychrophile.

**References**

[1] Gao M, et al. Fungal Lactamases: Their Occurrence and Function. Front. Microbiol. 2017, 8, 1775 :1-17.

[2] Glenn A. E. et al*.* Two Horizontally Transferred Xenobiotic Resistance Gene Clusters Associated with Detoxification of Benzoxazolinones by Fusarium Species. PloS one 2016, 11(1): e0147486. 10.1371/journal.pone.0147486.

[3] Pillai-Kastoori L, et al. A systematic approach to quantitative Western blot analysis. Anal. Biochem. 2020, 593, 113608.

